# Future medical student practice intentions: the South Africa experience

**DOI:** 10.1186/s12909-020-02361-5

**Published:** 2020-11-16

**Authors:** Amy Clithero-Eridon, Cameron Crandall, Andrew Ross

**Affiliations:** 1 Department of Family & Community Medicine, University of New Mexico, MSC 09-5040, 1 UNM, Albuquerque, NM 87131-0001 USA; 2 Department of Emergency Medicine, University of New Mexico, MSC 11-6025, 1 UNM, Albuquerque, NM 87131-0001 USA; 3 Department of Family Medicine, University of KwaZulu-Natal, 24 Jupiter Rd, Westville, Durban, 3629 South Africa

## Abstract

**Background:**

Primary care is a broad spectrum specialty that can serve both urban and rural populations. It is important to examine the specialties students are selecting to enter, future community size they intend to practice in as well as whether they intend to remain in the communities in which they trained.

**Aim:**

The goals of this study were to characterize the background and career aspirations of medical students. Objectives were to (1) explore whether there are points in time during training that may affect career goals and (2) assess how students’ background and stated motivations for choosing medicine as a career related to intended professional practice.

**Setting:**

The setting for this study was the Nelson R. Mandela School of Medicine, located in Durban, South Africa.

**Methods:**

We conducted a cross-sectional survey of 597 NRMSM medical students in their first, fourth, or sixth-year studies during the 2017 academic year.

**Results:**

Our findings show a noticeable lack of interest in primary care, and in particular, family medicine amongst graduating students. Altruism is not as motivating a factor for practicing medicine as it was among students beginning their education.

**Conclusion:**

Selection of students into medical school should consider personal characteristics such as background and career motivation. Once students are selected, local context matters for training to sustain motivation. Selection of students most likely to practice primary care, then emphasizing family medicine and community immersion with underserved populations, can assist in building health workforce capacity. There are institutional, legislative, and market pressures influencing career choice either toward or away from primary care. In this paper, we will discuss only the institutional aspects.

## Background

There is growing international momentum for medical schools to be socially accountable through research, service, and education [1, 2]. Social accountability carries an expectation that health professional graduates will obtain the competencies necessary to address the priority health needs of the communities they serve. Competencies beyond technical proficiency include a holistic approach to wellness that encompasses physical, mental, and social well-being as well as a community orientation to address broad social determinants of health. Physicians who may be best situated to deliver this care are primary care physicians and, in particular, family medicine (FM) physicians. The impact of FM to the people of Africa is best captured in the Statement of Consensus of Family Medicine in Africa [3]. This statement recognizes FM as integral to the district health system, where multi-level care capacity is critical to the achievement of equitable health outcomes for all. Family medicine physicians are taught to educate, treat and prevent disease at both an individual and a community level. They are expert generalists who are trained to care for the majority of health problems seen over the life span in both clinics and district hospitals. Persons with access to this specialty have better health outcomes with less cost to both the healthcare system and in downstream costs to patients [4, 5].

Training physicians to meet the needs of communities requires a multipronged approach beginning with student selection, continuing with placement in appropriate contexts, and emphasizing primary care throughout the curriculum. The Network Towards Unity for Health (THEnet) is a consortium of 13 universities that, in addition to subscribing to the principles of social accountability, are also gathering strong evidence on a global basis for what works in what context to achieve social accountability [6]. THEnet’s proposed methods for measuring progress towards meeting the educational component of this vision include examining socio-demographic and practice intention characteristics. Specific indicators include whether matriculating students selected into the training program demographically mirror the population served and whether graduating students choose to practice primary care and intend to practice in areas of high need such as underserved and rural areas [6].

While South Africa has a shortage of all health care specialties, an argument can be made that none is as critical as the need for primary care providers and, in particular, family medicine. Physicians [7, 8]. Africa has not historically had FM physicians, but several African countries, including Ghana, Botswana, Uganda, Kenya, and Nigeria, are recognizing the impact this specialty can have in improving the delivery of care and moving towards achieving universal healthcare [9]. In South Africa, FM was not recognized as a specialty until 2007. Universities now include this specialty in undergraduate and postgraduate training but it remains underrepresented [9, 10].

Characterization of medical student background and career aspirations may give insight into whether there are specific points during the training curriculum that influences the student’s choice of specialty and community of practice. The goals of this study were to characterize the background and career aspirations of medical students at the Nelson R. Mandela School of Medicine (NRMSM), located in Durban, South Africa. Objectives were to (1) explore whether there are points in time during training that may affect career goals and (2) assess how students’ background and stated motivations for choosing medicine as a career related to intended professional practice. We hypothesized that students’ personal characteristics would affect future practice intentions.

## Methods

We used a cross-sectional design to survey three cohorts of medical from the NRMSM distinguished by year of training (1st, 4th, and 6th year medical student, *N* = 597) to assess future career aspirations. We selected and adapted questions and indicators from the Training for Health Equity (THEnet) graduate outcome project for this study [6, 11]. Questions from the survey were multiple-choice responses. The study data were collected using an online survey for first-year students as part of a broader end of course evaluation. Paper-based surveys were used for 4th and 6th-year students. All data were collected by researchers at NRMSM and transmitted to investigators at the University of New Mexico, School of Medicine in Albuquerque, NM, United States (U.S.) [12].

### Ethical considerations

The Biomedical Research and Ethics Committee of the University of KwaZulu-Natal approved the study design (HSS/0119/017D). Each participant provided written informed consent before participation in the study.

### Setting

1st-year students completed the questionnaire online after their Becoming a Professional module. The module is a multidisciplinary course for first-year students to build foundational knowledge and skills emphasizing public health and community service [13]. All 4th-year medical students enrolled in the Community and Evidence-Based practice 111 module were asked to complete the questionnaire in September 2017. This module introduces students to population health by linking diseases within communities to social determinants of health [14, 15]. 6th year students completed the questionnaire after each seven-week FM rotation in 2017. All students were given an information sheet and signed consent prior to completing the questionnaire.

### Indicators

We selected questions from THEnet survey on specialty, intent to practice abroad, future community, and future community size to understand practice intentions and career aspirations. We selected questions regarding initial motivation for a career in medicine to see if there was a correlation to future practice. Selected background questions included language, parental education, ethnicity, gender, and aspects of disadvantage to see if students mirror the population served as well as ascertain if there is a correlation to future practice intention.

#### Specialty

We asked respondents what discipline they were most likely to pursue after graduation. We were primarily interested in characterizing future specialty decisions. The 22 respondents (22/597, 4%) that did not select a future specialty were excluded from the analysis. We grouped responses as primary care and non-primary care. For this study, we defined primary care physicians as general internal medicine physicians, family medicine physicians, and pediatricians [5]. We parsed out FM as a distinct category from primary care where appropriate. This question asked respondents to choose one response only. If more than two choices were selected, the response was changed to “don’t know” as some selected over five responses, which indicated they did not know what their primary discipline might be.

#### Migration

We asked respondents if, after completing their medical studies, they intended to work abroad. If they answered yes, they were given a selection of reasons for their response.

#### Community size

Respondents indicated what size of the community they grew up in as well as the size of the community they intend to practice immediately after graduation. We used the United Nations Demographic and Social Statistics recommended population classification [16]. We then categorized responses into populations less than 10,000, populations 10,000 to 99,999, and populations greater than 100,000.

#### Motivation

Respondents provided their motivation for choosing medicine as a career. Selections were: (1) To make a difference/help others; (2) Medicine is a good career (job/financial security); (3) To serve my community; (4) Medicine is interesting; (5) There is a need for more doctors in my country. We classified responses 1, 3 or 5, as altruistic motivations and 2 or 4 as intrinsic.

#### Language

Respondents indicated if they spoke any language other than English well enough to practice medicine. We grouped choices into English, Afrikaans, Indigenous dialect, or other languages. The survey asked respondents to select no more than three languages. If someone gave more than three, the first three written responses were selected for inclusion in the results.

#### Education

Respondents indicated the highest level of education attained by their mother and father as an indication of socioeconomic status. We then collapsed selections into unknown, missing, college and no college. Missing or unknown responses were combined for parental education.

#### Ethnicity

Students were grouped as Black or non-Black persons. Due to an error in survey distribution, ethnicity was omitted from the online survey used for year one respondents.

#### Gender

Collected in years 4 and 6 only. Due to an error in survey distribution, gender was omitted from the online survey used for year one respondents.

#### Self-identification

Students in years 1, 4, and 6 were asked if they identified as a member of one or more of the following: (1) Religious minority group; (2) Refugee; (3) Recent immigrant to South Africa (less than 5 years); (4) Disadvantaged caste group; (5) Black African; (6) Other underserved group; (7) None of the above. The Year 1 survey did not include Black African as an option. Consequently, we categorized affirmative responses in categories 1 through 4 or 6, as disadvantaged. If the response was 7, none of the above, then they were categorized as not disadvantaged.

### Data analysis

Data were entered into REDCap, an electronic database management system hosted at the University of New Mexico [17]. We analyzed the data in SAS (version 9.4) [18].

We summarized categorical responses within and between cohorts with proportions. To compare categorical variables between groups, we used the chi-square test or Fisher’s exact test (FET). We used the Newcombe-Wilson Hybrid Score [19] method to calculate confidence intervals for differences in proportions.

## Results

The overall response rate was 84%. The response rate from first-year students was higher than subsequent cohorts (1st year: 246/254 (97%); 4th year: 188/263 (71%); 6th year: 163/195 (84%)).Overall, there were slightly more female than male respondents. Nearly all respondents were 20 to 29 years old (98% 4th year; 97% 6th year). Over two-thirds were Black students (Table 1).

**Table 1:** General Characteristics

	YEAR 1***N*** = 246		YEAR 4 ***N*** = 188		YEAR 6 ***N*** = 163		Total ***N*** = 597	
	N	%	N	%	N	%	N	%
**Sex**								
*Male*	N/A		82	45%	56	35%	138	40%
*Female*	N/A		100	55%	104	65%	204	60%
**Race**								
*Black*	N/A		119	70%	115	72%	234	71%
*Non-Black*	N/A		52	30%	45	28%	97	29%
**Home Language**								
*English*	40	16%	60	32%	48	30%	148	25%
*Afrikaans*	0	0%	1	1%	2	1%	3	1%
*Indigenous*	179	74%	126	67%	111	69%	416	70%
*Other*	24	10%	1	1%	1	1%	26	1%
**Disadvantaged Background (as based on self-identification)**								
*Yes*	92	38%	137	24%	121	80%	350	61%
*No*	153	62%	44	76%	30	20%	227	39%
**Home Community Size (population)**								
*100,000+*	51	21%	63	41%	52	33%	166	30%
*10,000–99,999*	130	53%	51	34%	44	28%	225	41%
*< 10,000*	65	26%	38	25%	60	38%	213	29%
**Practice Community Size (population)**								
*100,000+*	87	35%	87	48%	62	39%	236	40%
*10,000–99,999*	127	52%	66	37%	41	26%	234	40%
*< 10,000*	32	13%	27	15%	55	35%	114	20%
**Future Specialty**								
*Primary Care*	73	30%	66	39%	57	35%	196	34%
*Family Medicine*	*33*	*13%*	*23*	*14%*	*6*	*4%*	*62*	*11%*
*Non-Primary Care*	143	58%	70	42%	68	42%	281	49%
*Undecided*	30	12%	32	19%	36	22%	98	17%

*N/A* Data not available

*Source:* Authors’ own work

### Language concordance

Most respondents spoke English as a second language. Slightly more than two-thirds of respondents (70%) were able to communicate with patients using an indigenous dialect. Few spoke Afrikaans.

### Future specialty

Overall, about one-third (34%) of respondents indicated an intention to go into primary care, with about one-third of these respondents (62/196, (32%)) stating an intention to go into FM. Intention to go into FM varied by cohort.

Among 4th and 6th year respondents, a similar proportion of Black (80/216, 37%) and non-Black (35/94, 37%) students selected primary care as their future specialty. Within those who selected primary care, more Black students selected FM as their future specialty (26%) compared to non-Black students (14%). However this difference was not significant (difference (Δ): 12%; 95% confidence interval (CI): − 3, 27%).

A similar proportion of female (66/184 (36%) and male respondents (47/123, 38%) indicated a plan to enter primary care. Among respondents selecting primary care, a similar proportion of female (14/66, 21%) and male (11/47, 23%) respondents indicated a plan to enter FM. More Black males selected FM (11/37, 30%) than non-Black males (0/10, 0%) (Δ: 30%; CI: − 1, 46%). A similar proportion of Black females (9/41, 22%) and non-Black females (5/25, 20%) selected FM.

Black respondents were more decisive than non-Black respondents regarding their future specialty. A higher proportion of non-Black respondents (30/94, 32%) did not know what specialty they would select after graduation compared to Black respondents (35/216, 16%) (Δ: 16%; CI: 6, 27%).

Parental educational status did not affect whether a respondent intended to enter primary care or not in any of the cohorts.

### Motivation for medicine and the effect on future specialty (Table 2)

Overall, those preferring primary care were more likely to be classified as altruistic (78%) compared to non-primary care (58%) (Δ: 20%; CI: 12, 27%). Between cohorts, the percentage of students entering primary care remained constant (1st year: 30%, 4th year: 39%, 6th year: 35%, x^2^ = 1.89, df = 2, *p* = 0.389, Table 1) but FM decreased in later cohorts (1st year: 13%, 4th year: 14%, 6th year: 4%, x^2^ = 10.99, df = 2, *p* = 0.002, Table 1). Among respondents indicating primary care, the effect of altruism as a motivating factor was highest among the 1st year students and lower in subsequent cohorts (1st year: 93%, 4th year: 76%, 6th year: 63%, x^2^ = 18.05, df = 2, *p* < 0.001, Table 2).

**Table 2:** Motivation for medicine and the effect on future specialty

	Total		Primary Care		Family Medicine		Non-Primary Care		Don’t Know	
	N	%	N	%	N	%	N	%	N	%
Total										
Altruistic	435	67%	153	78%	53	85%	214	58%	68	76%
Intrinsic	217	33%	42	22%	9	15%	153	42%	22	24%
Year 1										
Altruistic	216	63%	68	93%	31	94%	125	52%	23	79%
Intrinsic	127	37%	5	7%	2	6%	116	48%	6	21%
Year 4										
Altruistic	120	76%	50	76%	17	74%	47	78%	23	74%
Intrinsic	37	24%	16	24%	6	26%	13	22%	8	26%
Year 6										
Altruistic	99	65%	35	63%	5	83%	42	64%	22	73%
Intrinsic	53	35%	21	38%	1	17%	24	36%	8	27%

*Source:* Authors’ own work

### Relationship of intended community size, home community size, and future specialty, by cohort. Where did they come from and where are they going and what motivates them (Table 3)

There was no relationship of home community size or intended practice community size and specialty intention in any cohort. We also looked at the change in community size (home vs. practice community). In all cohorts, students stated an intention to stay in the same size community or one more urban. This statement remains true, regardless of whether students reported they are from a disadvantaged background. We did not see any significant differences by specialty or cohort.

**Table 3:** Intended community size compared to home community size and future specialty

	Less Urban		Same Size		More Urban		Total
	N	%	N	%	N	%	
**Year 1**							
*Primary Care*	12	16%	29	40%	32	44%	73
*Family Medicine*	9	27%	11	33%	13	39%	33
*Non-Primary Care*	16	11%	60	42%	67	47%	143
*Undecided*	6	20%	9	30%	15	50%	30
**Year 4**							
*Primary Care*	12	21%	22	39%	23	40%	57
*Family Medicine*	4	21%	6	32%	9	47%	19
*Non-Primary Care*	12	19%	29	46%	22	35%	63
*Undecided*	4	14%	18	64%	6	21%	28
**Year 6**							
*Primary Care*	4	8%	23	43%	26	16%	53
*Family Medicine*	0	0%	5	83%	1	17%	6
*Non-Primary Care*	13	20%	23	35%	30	45%	66
*Undecided*	6	18%	17	50%	11	32%	34

*Source:* Authors’ own work

### Staying or leaving?

Less than one-third of first-year students (27%) indicated they would go abroad after graduation. Similarly, 28% of 4th-year and 31% of 6th-year students indicated they were going to go abroad after their medical studies. Among 4th and 6th year students who indicated an intention to work abroad, the primary reason for leaving was to gain experience (4th: *n* = 28, 54%; 6th: n = 28, 56%) followed by the belief there were better opportunities overseas (4th: *n* = 14, 27%; 6th: *n* = 12, 24%).

Among 4th and 6th year students who indicated an intention to stay in South Africa, the reasons for staying were the belief that their country needs doctors (4th: *n* = 32, 48%; 6th: *n* = 29, 45%) and a preference to stay home close to home and family (4th: n = 32, 46%; 6th: *n* = 25, 38%).

Black African respondents were significantly less likely to state an intention to work abroad than other respondents (Black: 25%, non-Black: 39%, Δ: − 14%; 95% CI: − 25, − 3%). We did not see a significant association of parental education with an intention to work abroad (mother with college: 28% intend to work abroad vs mother without college: 29% (Δ: − 1%; 95% CI: − 8, 7%); father with college: 30% intend to work abroad vs father without college: 27% (Δ: 3%; 95% CI: − 5, 10%)).

## Discussion

A substantial number of studies demonstrate that medical school admissions should consider personal characteristics such as background, specialty intention, and motivation for a career in medicine, as these can predict future practice patterns [11, 20, 21]. Community immersion experiences and exposure to rural locations and vulnerable populations are also deciding factors for student practice decisions [22–26]. NRMSM medical students identifying as from a disadvantaged background were not more likely to select a less urban community size for future practice, instead preferring the same size community or a more urban one than their home community.

There was language concordance with the population as the most common languages within the KwaZulu Natal province are IsiZulu, isiXhosa languages, and English [27]. The ability of physicians to speak the same language as their patients is important for clarity and shared understanding. For medical conditions, this is a crucial consideration. Patients who can understand their medical condition, able to follow directions, and, perhaps most importantly, ask questions have a better chance of improved healing and overall health. Language concordance promotes health equity by addressing disparities in care [28, 29]. The ability of physicians to speak the same language as their patients also connect the healthcare provider to the community served.

Equitable distribution of physicians is a top priority for the South African Department of Health [30], as nearly half of the population lives in rural areas. Yet, rural communities have access to only 12% of the country’s doctors [8]. Although there is strong evidence both locally and internationally that students from a rural locale are more likely to work as health care professionals in rural areas [11, 21, 31–34], no South African health science university admission criteria favor rural students. Some policies (e.g., the national benchmark exam) actually disadvantage rural students as they may not have as strong an educational background as their urban peers. Currently, South African training institutions use race as a proxy for social accountability to enable universities to meet training number goals with the expectation that this will assist in achieving socially responsible needs for the country [35]. South African medical schools use both academic and non-academic criteria when reviewing applicants. The NRMSM selects students based on identifiable markers such as race and socioeconomic background to mirror the general population. Student selection based on race is in line with South African mandates [27]. NRMSM accepts 250 students per year, with about one-third selected from the most socio-economically disadvantaged schools (quintiles 1 and 2) without racial quotas [35]. Half of all students are selected on merit, with 20% reserved for students with prior higher education. With regards to ethnicity, 69% of places are reserved for Black students, 19% for Indian, 9% for mixed race, 2% for White and 1% for other races [35].

Once students are selected, local context matters for training. Providing training within communities helps students build a connection to the community making it more likely they will return after graduation [36]. Community experiences help build confidence in clinical skills as well as developing empathy for patients as persons, not just patients by making the connection between classroom learning and actual practice [8, 9]. Knight et al. showed that students chose to return to their home or similarly sized community during their community immersion modules [14]. However, for rural students, this represented only 39% of students.

Our findings showed no difference in intention to enter primary care based on ethnicity or gender. There is a difference in student intention to enter FM based on ethnicity and gender with more male Black students choosing this as their future FM practice across cohorts; however, this effect decreased in students nearing graduation. There are numerous reasons supply and demand of physicians are unequal, and students choose, or do not choose, FM for multiple reasons [37]. Medical schools can assist in alleviating physician shortages through curricular interventions as there are various factors that affect practice intention [38, 39]. Selection of students most likely to practice primary care, then emphasizing FM and community immersion with underserved populations, might assist in building this needed specialty (currently only 1000 for a population of 55 million) [40]. There is clearly a need for FM to be at the forefront of medical education for students to consider it as a future specialty as well as influence policy and health system direction [41].

Intention to practice in a rural and underserved area is also important. Strong attachment to home community and commitment to living rural impacts selection of specialty [42]. Our findings suggest that altruistic motivation may be a factor for studying medicine at the beginning of medical school that is not sustained in subsequent years. Altruistic reasons for entering medicine also seemed to impact student preference for primary care, FM, and serving in a less urban area. Because it has been documented that altruism decreases within the curricular experience, sustaining this altruism in subsequent years to remind students and reinforce intentions is needed so graduating students do not shift to intrinsic drivers or forget their original motivation [43]. Reasons for decreased altruism amongst final year students at NRMSM might be that they are 5 years removed from their first community immersion experience working with community-based organizations as their mentors [44]. While we did not specifically investigate curricular components, the phenomenon of the hidden curriculum [45], both positive and negative, should be considered as a potential influencer on both student specialty choice and inner motivations. Perceptions of FM as a career and the overall image of FM can be reinforced by peers and professors as well as by societal rewards and recognition of FM as respected career [46, 47]. Conversely since this is not a longitudinal cohort study, NRMSM may be selecting and immersing students to a better degree than 6 years ago as demonstrated by the year one cohort that is altruistic, interested in primary care and not intending to go abroad as compared to cohort six who received their education at an earlier point in time before social accountability and community immersion became prominent within the curriculum.

A significant number of students within each cohort were uncertain or intended to leave South Africa. Identifying these students and then immersing them within communities may encourage them to remain within South Africa, particularly amongst those students who seemed to understand that more physicians are needed in South Africa. It is not apparent if reasons for leaving are based more on South African economic reality than other reasons. With almost 30% of graduates planning to leave South Africa after graduation, interest in FM as a specialty may be affected. Our findings showed that Black African students were less likely to state an intention to work abroad than non-Black students and is an indicator of social accountability success [6]. We did not observe any association between parental education, used as an indicator of socioeconomic status, and an expressed intention to leave. Portions of our findings did not correlate with a more extensive longitudinal cohort study that found students from backgrounds of higher income, including students from Walter Sisulu University in South Africa, were more likely to go abroad after graduation [11]. Findings may also differ as Walter Sisulu University is part of THEnet consortium of health professional organizations that subscribe to principles of social accountability (although NRMSM has recently joined) and base their curriculum on these values and community immersion experiences and specifically train their students for the specialties their community needs [42].

### Limitations

This study reflects a very local reality of a country where FM was not recognized as a specialty until 2007 as compared to countries where FM has not developed as a discipline at all or those who recognized it 30–40 years ago. Due to an error in the online survey distribution, we did not collect complete demographic data for first-year students. We do not believe that this omission significantly impacted our findings as the gender and ethnicity of the first year students overall was similar to other years. Also, responses are self-reported intentions rather than actual outcomes as there is no formal mechanism for surveying graduates after they leave the school. This makes it impossible to compare student intention to reality. Another limitation was some respondents selected more than the minimum response required for future specialty and languages spoken. Definitions for altruistic and intrinsic were based on construct validity and were not formally validated.

## Conclusion

In South Africa, half of the population lives in rural areas with access to only 12% of the country’s doctors is in critical need of family physicians willing to treat underserved populations which is an issue that cannot be solved by curricular changes only, however it can be a piece of the solution. Graduates of NRMSM enter with altruistic motivations and a value system that orient them for careers as primary care physicians. Sustaining initial motivations through community immersion with mentors who reinforce the values of primary care and community involvement throughout the curriculum with particular emphasis during the final years when burnout and loss of empathy are more likely to occur is needed. Identification of matriculating and graduating medical students’ practice intentions, especially whether they intend to practice in an underserved area, can give universities as well as broader workforce planners and policymakers’ information on how training might be designed to retain first-line health practitioners in areas of need. Family medicine should continue to be emphasized throughout the curriculum as a discipline that improves health equity and access to care. As proposed by Rodriguez, inclusion of FM providers in leadership roles may also be a strategy to influence perceptions of this specialty as a prestigious career choice [47]. Students who are uncertain about their future specialty and students who identify primary care and specifically FM as their career choice should be mentored to develop and maintain interest throughout their training.

## Data Availability

The datasets generated and/or analysed during the current study are not publicly available as they ware part of a larger study on social accountability. However, the specific information used to generate the findings in this study are available from the corresponding author on reasonable request.

## Abbreviations

FM: Family Medicine
THEnet: The Network Towards Unity for Health
NRMSM: Nelson R. Mandela School of Medicine
U.S.: United States

## References

[1] Boelen C, Heck JE, Health WHOD of D of HR for (1995). Defining and measuring the social accountability of medical schools.

[2] Boelen C, Dharamsi S, Gibbs T (2012). The social accountability of medical schools and its indicators. Educ Health.

[3] Mash RB, Reid S (2010). Statement of consensus on family medicine in Africa: conference report. Afr J Prim Health Care Fam Med.

[4] Starfield B (2012). Primary care: an increasingly important contributor to effectiveness, equity, and efficiency of health services. SESPAS report 2012. Gac Sanit.

[5] Starfield B, Shi L, Macinko J (2005). Contribution of primary care to health systems and health. Milbank Q.

[6] Larkins SL, Preston R, Matte MC (2013). Measuring social accountability in health professional education: development and international pilot testing of an evaluation framework. Med Teach.

[7] Willcox ML, Peersman W, Daou P (2015). Human resources for primary health care in sub-Saharan Africa: progress or stagnation?. Hum Resour Health.

[8] Hatcher AM, Onah M, Kornik S (2014). Placement, support, and retention of health professionals: national, cross-sectional findings from medical and dental community service officers in South Africa. Hum Resour Health.

[9] Mash R, Howe A, Olayemi O (2018). Reflections on family medicine and primary healthcare in sub-Saharan Africa. BMJ Glob Health.

[10] Hellenberg DA, Gibbs T, Megennis S (2005). Family medicine in South Africa: where are we now and where do we want to be?. Eur J Gen Pract.

[11] Larkins S, Johnston K, Hogenbirk JC, et al. Practice intentions at entry to and exit from medical schools aspiring to social accountability: findings from the training for health equity network graduate outcome study. BMC Med Educ. 18Epub ahead of print December 2018. 10.1186/s12909-018-1360-6. 10.1186/s12909-018-1360-6 PMC6234627 30424760

[12] School of Medicine | The University of New Mexico, https://hsc.unm.edu/school-of-medicine/ (Accessed 23 July 2019).

[13] Becoming a Professional, http://mepi.ukzn.ac.za/MEPIComponents/Public-Health/Becoming-a-Professional.aspx (Accessed 22 Dec 2019).

[14] E Knight S, J Ross A, Mahomed O (2017). Developing primary health care and public health competencies in undergraduate medical students. S Afr Fam Pract.

[15] Ali RO, Ross AJ, Nkabinde TC (2019). Knowledge of final-year medical students at the University of KwaZulu-Natal about family medicine, and long-term career choices. S Afr Fam Pract.

[16] United Nations Statistics Division - Demographic and Social Statistics, https://unstats.un.org/unsd/demographic/sconcerns/densurb/densurbmethods.htm (Accessed 29 Dec 2019).

[17] Harris PA, Taylor R, Thielke R (2009). Research electronic data capture (REDCap)—a metadata-driven methodology and workflow process for providing translational research informatics support. J Biomed Inform.

[18] SAS Institute Inc. SAS 9.4 Companion for Windows.

[19] Newcombe RG (1998). Interval estimation for the difference between independent proportions: comparison of eleven methods. Stat Med.

[20] Playford D, Ngo H, Gupta S (2017). Opting for rural practice: the influence of medical student origin, intention and immersion experience. Med J Aust.

[21] Rabinowitz HK, Diamond JJ, Markham FW (2012). The relationship between entering medical Studentsʼ backgrounds and career plans and their rural practice outcomes three decades later. Acad Med.

[22] Strasser RP, Lanphear JH, McCready WG (2009). Canada’s new medical school: the northern Ontario School of Medicine: social accountability through distributed community engaged learning. Acad Med.

[23] Strasser R, Neusy A-J (2010). Context counts: training health workers in and for rural and remote areas. Bull World Health Organ.

[24] Reeve C, Woolley T, Ross SJ (2017). The impact of socially-accountable health professional education: a systematic review of the literature. Med Teach.

[25] Barrett FA, Lipsky MS, Nawal Lutfiyya M (2011). The impact of rural training experiences on medical students: a critical review. Acad Med.

[26] De Vries E, Reid S (2003). Do south African medical students of rural origin return to rural practice?. S Afr Med J.

[27] StatsSA | Community Survey 2016 Provincial profile: KwaZulu-Natal, 2016, http://cs2016.statssa.gov.za/?portfolio_page=community-survey-2016-provincial-profile-kwazulu-natal-2016 (Accessed 24 Sep 2018).

[28] Quigley DD, Elliott MN, Hambarsoomian K (2019). Inpatient care experiences differ by preferred language within racial/ethnic groups. Health Serv Res.

[29] Hasnain-Wynia R, Wolf MS (2010). Promoting health care equity: is health literacy a missing link?. Health Serv Res.

[30] National Department of Health. Human Resources for Health for South Africa 2030 | Health systems trust. Health Systems Trust, http://www.hst.org.za/publications/human-resources-health-south-africa-2030 (Accessed 6 June 2017).

[31] Couper ID, Hugo JFM, Conradie H (2007). Influences on the choice of health professionals to practice in rural areas. South Afr Med J Suid-Afr Tydskr Vir Geneeskd.

[32] Poole P, Stoner T, Verstappen A (2016). Medical students: where have they come from; where are they going?. N Z Med J.

[33] Kent M, Verstappen AC, Wilkinson T, et al. Keeping them interested: a national study of factors that change medical student interest in working rurally. Epub ahead of print 8 October 2018. DOI: 10.22605/RRH4872. 10.22605/RRH4872 30293435

[34] Versteeg M, du Toit L, Couper I (2013). Building consensus on key priorities for rural health care in South Africa using the Delphi technique. Glob Health Action.

[35] Van der Merwe LJ, Van Zyl GJ, Gibson ASC (2016). South African medical schools: current state of selection criteria and medical students’ demographic profile. S Afr Med J.

[36] Ross BM, Daynard K, Greenwood D (2014). Medicine for somewhere: the emergence of place in medical education. Educ Res Rev.

[37] Strasser R, Kam SM, Regalado SM (2016). Rural health care access and policy in developing countries. Annu Rev Public Health.

[38] Reid SJ, Couper ID, Volmink J (2011). Educational factors that influence the urban-rural distribution of health professionals in South Africa: a case-control study. South Afr Med J Suid-Afr Tydskr Vir Geneeskd.

[39] Rohan-Minjares F, Alfero C, Kaufman A (2015). How medical schools can encourage students’ interest in family medicine. Acad Med.

[40] Moosa S, Peersman W, Derese A (2018). Emerging role of family medicine in South Africa. BMJ Glob Health.

[41] Mash R, Ogunbanjo G, Naidoo SS (2015). The contribution of family physicians to district health services: a national position paper for South Africa. S Afr Fam Pract.

[42] Pálsdóttir B, Barry J, Bruno A (2016). Training for impact: the socio-economic impact of a fit for purpose health workforce on communities. Hum Resour Health.

[43] Colliver JA, Conlee MJ, Verhulst SJ (2010). Reports of the decline of empathy during medical education are greatly exaggerated: a reexamination of the research. Acad Med.

[44] van Wyk JM, Knight SE, Dlungwane T (2016). Developing social accountability in 1st-year medical students: a case study from the Nelson R Mandela School of Medicine, Durban, South Africa. Afr J Health Prof Educ.

[45] Hafferty FW (1998). Beyond curriculum reform: confronting medicine’s hidden curriculum. Acad Med J Assoc Am Med Coll.

[46] Erikson CE, Danish S, Jones KC (2013). The role of medical school culture in primary care career choice. Acad Med J Assoc Am Med Coll.

[47] Rodríguez C, López-Roig S, Pawlikowska T (2015). The influence of academic discourses on medical students’ identification with the discipline of family medicine. Acad Med J Assoc Am Med Coll.

